# Does Childhood Trauma Predict Impulsive Spending in Later Life? An Analysis of the Mediating Roles of Impulsivity and Emotion Regulation

**DOI:** 10.1007/s40653-023-00600-7

**Published:** 2024-01-05

**Authors:** Thomas Richardson, Annelise Egglishaw, Monica Sood

**Affiliations:** School of Psychology, University of Southampton, Building 44, Highfield Campus, University Road, Southampton, SO17 1BJ England, UK

**Keywords:** Adverse childhood experiences, Childhood trauma, Impulsive spending, Impulsivity, Emotion dysregulation

## Abstract

We sought to investigate whether adverse childhood experiences increase impulsive spending in later life, and whether emotion dysregulation and impulsivity mediate this association. Limited research has examined associations between these factors, and examining the mechanisms involved may inform interventions for impulsive spending. This study used a cross-sectional, correlational design including 189 adult participants who completed an online survey assessing childhood trauma, adverse childhood experiences, impulsive spending, impulsivity, and emotion dysregulation. Greater adverse childhood experiences and childhood trauma were positively correlated with impulsive spending, as well as general impulsivity and emotion dysregulation. Mediation analyses indicated that emotion dysregulation and greater impulsivity accounted for the positive relationship between childhood trauma and impulse spending. Adverse childhood experiences and childhood trauma are associated with increased risk of impulse spending in adulthood via elevated general impulsivity and emotion dysregulation.

## Introduction

*Adverse childhood experiences* (ACEs) are potentially traumatic events that could impact a child’s health and development (Boullier & Blair, 2018). Between 2020 and 2021, there were over 24,800 child abuse offences reported in England and Wales, an 11.6% increase compared to 2019 and 2020 (Statista Research Department, 2021). There are five main types of ACEs, including physical abuse, emotional abuse, sexual abuse, physical neglect, and emotional neglect (Gilbert et al., 2009). ACEs also refer to household dysfunction, such as parental separation, mental illness in the family, domestic violence, and an incarcerated family member (Boullier & Blair, 2018). It should be acknowledged that adversity may come in different forms and durations and, therefore, ACEs alone may not provide a comprehensive list of childhood trauma (Boullier & Blair, 2018). Research examining the impact of ACEs has found associations with poorer health outcomes in later life, these being both physical and mental (Boullier & Blair, 2018). Further to this, ACEs are believed to impact behaviour, life opportunities, and economic stability (Boullier & Blair, 2018). The link between ACEs and economic stability is pertinent to this research, which investigates the impact of ACEs on *impulse spending* in later life.

Impulse spending describes when “a consumer experiences a sudden, often powerful and persistent urge to buy something immediately” (Rook, 1987, p. 191). Valence et al. (1988) have suggested three constructs involved in the related concept of compulsive spending: (a) strong emotional activation (an increase in psychological tension), (b) high cognitive control (knowledge that spending will reduce this tension), and (c) high reactivity (a preference for tension reduction rather than a solution to the problem). At present, there is limited research investigating an association between ACEs and impulse spending, though researchers have discovered that ACEs are associated with gambling in later life; Lotzin et al. (2018) found that four out of five gamblers reported experiencing at least one ACE in their lifetime, indicating that ACEs increase the likelihood of becoming a problem gambler by 60%.

*Impulsivity* is defined as “actions that are poorly conceived, prematurely expressed, unduly risky, or inappropriate to the situation which often results in undesirable outcomes” (Evenden, 1999, p. 348). Whiteside and Lynam (2001) suggested that certain personality traits are associated with impulsivity, such as urgency, lack of premeditation, lack of perseverance, and sensation seeking. Research shows that high exposure to ACEs is related to impulse control difficulties in later life, specifically negative urgency (an impulsive act used to improve one’s mood, without thought of the later consequences (Shin et al., 2018)). Youn and Faber (2000) have suggested that a lack of control, or impulsive attitude, is a potential contributing factor to impulse spending. Bratko et al. (2013) found that trait impulsivity positively correlated with impulse spending. Research has also shown that specific trait impulsivity elements of cognitive complexity and motor impulsivity predict impulsive spending (Alloway et al., 2016). Other research has shown a relationship with non-planning, attention, and motor impulsivity (Sokić et al., 2020). Together, this evidence indicates that impulsivity may be linked to ACEs and impulse spending, and that those who act impulsively may also struggle to regulate their emotions.

*Emotional dysregulation* is defined as “the impaired ability to regulate and/or tolerate negative emotional states” (Dvir et al., 2014, p. 1). There are various processes involved in emotional regulation, including biological, psychological, and interpersonal mechanisms (Ford, 2005). Emotion regulation involves the ability to control how and when emotions are felt as well as the intensity with which emotions are experienced (Dvir et al., 2014). Research shows that a large majority of individuals who spend impulsively report feeling better after their purchases (Gardner & Rook, 1988). Iyer et al. (2020) contended that a consumer’s mood may act as an explanation for the affective and cognitive processes involved in impulse spending, suggesting that there is an emotional aspect that contributes to a need to spend impulsively. In line with this, in a study using retrospective reporting, Ozer and Gultekin (2015) found that mood prior to making a purchase was linked to increases in the likelihood of impulsive spending.

There is little research linking childhood adversity and impulsive spending in adulthood. One study found that increased negative mood regulation expectancies, alexithymia, and childhood maltreatment were all independent predictors of compulsive buying (Kaur & Mearns, 2021). This study also showed that negative mood regulation expectancies moderated the effect of maltreatment on impulse spending (Kaur & Mearns, 2021).

A small body of literature has investigated the role of both trait impulsivity and emotional dysregulation as overlapping and interacting risk factors for other impulsive behaviours. For example, Jakubczyk et al. (2018) found that those with alcohol use disorders had greater impulsivity via emotional dysregulation. Research on a brief intervention for risky behaviours showed that reductions in risky behaviours were linked to changes in emotional dysregulation rather than impulsivity (Weiss et al., 2015). However, no research to date has examined the role of both impulsivity and emotional dysregulation together in predicting compulsive spending and their relationship with childhood trauma.

In the present study, we sought to investigate the link between childhood trauma and impulsive financial behaviours in later life, and whether this association is accounted for by impulsivity and emotional dysregulation. Little research has investigated the proposed relationships; examining the mechanisms through which ACEs impact impulsive spending is important to extend our understanding of the association between these two constructs and develop possible interventions. We examined three hypotheses: (a) greater childhood trauma will be correlated with more severe impulse spending in later life, (b) emotion dysregulation will mediate the relationship between ACEs and impulse spending, (c) greater impulsivity will mediate the relationship between ACEs and impulse spending.

## Method

### Participants

This research included a sample of 243 participants from the general population who were recruited online as well as students participating in exchange for university course credit. Non-university participants were given the opportunity to enter a prize draw to win a £50 voucher for participation. There were no inclusion criteria beyond being aged 18 years and above. Those with or without issues related to impulsive spending, childhood trauma, and mental health were eligible to participate. Ages ranged from 18 to 70 (*M* = 30.97, *SD* = 13.83). Most participants were female, aged between 18 and 25 years, and identified as White (see Table 1). Ninety-four participants reported a mental health condition; bipolar disorder was the most common, followed by anxiety and depression (see Table 2).

**Table 1:** Demographic Information

Category	Sub-Category	Frequency	Percent
Sex	Male	44	23.30
	Female	141	74.60
	Other	2	1.06
	Prefer not to say	2	1.06
Age	18–25	95	50.26
	26–35	39	20.63
	36–45	23	12.17
	46–55	11	5.82
	56 and above	21	11.11
Ethnicity	White	164	86.77
	Black/African American	13	6.88
	Asian	5	2.65
	Other	6	3.17
	Prefer not to say	1	0.53
Mental Health Condition	Yes	94	49.74
	No	89	47.10
	Prefer not to say	6	3.17

**Table 2:** Participant Mental Health Conditions

Mental Health Condition	Frequency	Percent
Depression	21	11.11
Anxiety	31	16.4
PTSD	8	4.23
Bipolar	46	24.3
OCD	7	3.7
Eating Disorder	2	1.0

*PTSD* post-traumatic stress disorder, *OCD* obsessive–compulsive disorder

### Measures

#### Childhood Maltreatment

To assess childhood maltreatment, the Childhood Trauma Questionnaire (CTQ; Bernstein et al., 1994) was used. This is a 28-item, retrospective, self-report tool that measures physical abuse, emotional abuse, sexual abuse, physical neglect, and emotional neglect during childhood. The scale also assesses aspects of the child’s environment growing up. Ten items were reverse coded. An example question is: “When I was growing up, my parents were too drunk or high to take care of the family”. Participants were asked to respond using a five-point Likert scale (1 = *never true* to 5 = *very often true*) to indicate the frequency of these scenarios. Scores were totalled; higher scores indicate greater exposure to childhood trauma. Cronbach’s alpha yielded excellent internal consistency (*α* = .94).

#### Impulsivity

To assess impulsivity, the Barratt Impulsivity Scale short form (BIS-11; Patton et al., 1995) was used. This scale is a 14-item, self-report tool used to assess impulsive or non-impulsive behaviours. Specifically, it assesses the personality and behavioural aspects of impulsivity. Six items were reverse coded. Participants responded to questions using a four-point Likert scale (1 = *rarely/never*, *4* = *almost always/always*). An example question is: “I squirm at plays or lectures”. Higher scores indicate greater impulsivity. Internal consistency in the current sample was good (*α* = .89).

#### Impulse Spending

To assess impulse spending, the Compulsive Buying Scale (CBS; Valence et al., 1988) was used. This is an 11-item, self-report tool used to assess spending habits. Participants responded to questions using a five-point Likert scale (1 = *strongly disagree*, 5 = *strongly agree*). An example question is: “When I have money, I cannot help but spend part or all of it”. A higher total score indicates a greater tendency towards impulse spending. Internal consistency in the current sample was excellent (*α* = .94).

#### Emotion Dysregulation

To assess emotion dysregulation, the Difficulties in Emotion Regulation Scale (DERS-16; Bjureberg et al., 2016) was used. This scale is a 16-item, self-report tool used to assess levels of difficulty with emotion regulation. Participants responded to questions using a five-point Likert scale (1 = *almost never*, 5 = *almost always*). An example question is: “I have difficulty making sense out of my feelings”. Higher scores indicate greater emotion regulation difficulties. Internal consistency in the current sample was excellent (*α* = .96).

Subscale scores were not computed as we sought to examine overall patterns and limit the number of analyses performed.

### Procedure

**Fig. 1:**
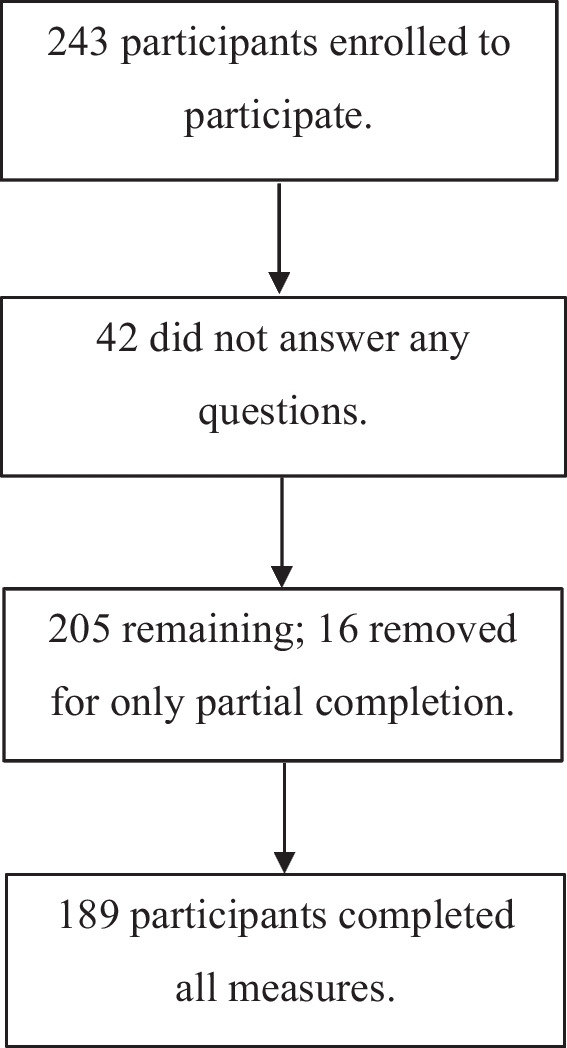
Flowchart of participation

Participants completed an online survey via Qualtrics. Ethical approval was obtained through the University of Southampton ethics committee. Participants took part in exchange for psychology course credit or were recruited online via social media and organisations for mental health and financial difficulties (which publicised the study though their social media accounts and email lists). All participants gave informed consent. Participants with mental health conditions were included and asked to provide further information regarding their diagnosis, if they wished to do so. See Fig. 1 for a recruitment flow chart.

### Design and Statistical Analysis

This research utilised a cross-sectional, correlational design to explore the link between ACEs and financial impulsivity in later life.

A one-tailed Pearson correlation was carried out to investigate whether childhood trauma correlated with impulse spending. Normal distribution was determined through visual inspection of histograms and statistics for kurtosis and skewness which were in the normal range (-2 to + 2) for all standardised measures. A parallel mediation analysis (using PROCESS v.4.0, Hayes, 2018) was conducted with childhood trauma as the independent variable, impulsive spending as the dependent variable, and general impulsivity and emotional dysregulation as parallel mediators. Given that some participants had a mental health diagnosis, we reconducted the mediation in the clinical and non-clinical groups separately to examine whether this impacted the results. Kline (2023) recommends 10 to 20 participants per parameter, indicating our sample size was sufficient for the mediation analyses and when divided by clinical vs. non-clinical participants.

## Results

A one-tailed Pearson’s correlation was used to assess the relationship between all five scales (see Table 3). Childhood trauma (CTQ) was positively correlated with impulsivity, impulsive buying, and emotion dysregulation (*p* < .001).

**Table 3:** Pearson’s Correlation Coefficients

Scale	CTQ	BIS-11	CBS
CTQ			
BIS-11	.46**	-	
CBS	.40**	.67**	-
DERS-16	.38**	.62**	.59**

*CTQ* Childhood Trauma Questionnaire, *BIS-11* Barratt Impulsivity Scale, *CBS* Compulsive Buying Scale, *DERS-16* Difficulty in Emotion Regulation Scale

^**^Correlation is significant at .01 level (1-tailed)

**Fig. 2:**
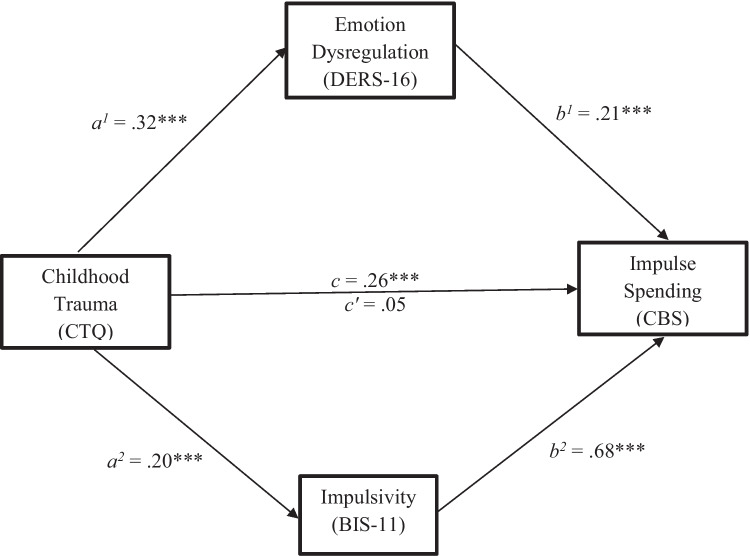
Parallel Mediation Model for the effect of Childhood Trauma on Impulse Spending via Emotion Dysregulation and Impulsivity. *Note*. Path *c’* = direct effect; path *c* = total effect. Estimated path coefficients are unstandardized. *** *p* < .001

The parallel mediation results (see Fig. 2) showed that the association between childhood trauma and impulsive spending was mediated by both impulsivity (*ab* = 0.14, *SE* = 0.03, 95% CI = [.08, .20]) and emotion dysregulation (*ab* = 0.07, *SE* = 0.02, 95% CI = [.03, .12]). Greater childhood trauma predicted greater impulsivity and emotional dysregulation which, in turn, predicted greater impulsive spending.

Given the high proportion of the sample with a mental health history (49.74%, *n* = 94), we re-ran the mediation analysis separately for the clinical and non-clinical participants. The indirect effects remained for non-clinical participants for both impulsivity (*ab* = .08, *SE* = 0.04, 95% CI = [.02, .16]) and emotional dysregulation: (*ab* = 0.09, *SE* = 0.05, 95% CI = [.02, .21]. For the clinical group there was an indirect effect via impulsivity (*ab* = 0.14, *SE* = 0.04, 95% CI = [.06, .24]), but not via emotional dysregulation (*ab* = 0.02, *SE* = 0.02, 95% CI = [-.01, .06]).

## Discussion

This study sought to examine the relationship between childhood trauma and impulse spending in later life, and the possible mediating roles of impulsivity and emotional dysregulation. Childhood trauma showed a weak but significant positive correlation with impulsive spending, indicating that greater exposure to childhood trauma increases the likelihood that an individual will impulsively spend in later life, supporting Hypothesis A. This aligns with previous findings showing that childhood maltreatment is linked to greater compulsive spending (Kaur & Means, 2021).

The parallel mediation results showed that emotion dysregulation and impulsivity mediated the relationship between childhood trauma and impulsive spending; specifically, greater childhood trauma predicted increased impulsive spending via greater impulsivity and emotional dysregulation (supporting Hypotheses B and C). These findings align with Espeleta et al. (2018) who found that emotion dysregulation mediated the association between childhood adversity and tendencies to engage in maladaptive behaviours. While their findings are not specific to impulse spending specifically, they showed that ACEs are linked to emotion dysregulation which may lead to difficulties in later life across a range of domains. The current findings also align with Shin et al. (2018) who showed that ACEs are related to impulsivity in later life, as well as Kaur and Mearns (2021) who found that negative mood regulation expectancy moderated the link between childhood adversity and impulsive spending. These findings are also consistent with research showing that mood prior to spending increases the likelihood of impulsive spending (Ozer & Gultekin, 2015).

No research prior to the current study has looked at impulsivity as a mediator between ACEs and impulse spending. The present research, for the first time, indicates that all factors are linked, and that emotion dysregulation and impulsivity are mechanisms involved in the relationship between ACEs and impulse spending. Whilst previous research has shown an interaction between impulsivity and emotional dysregulation in other risky impulsive behaviours such as alcohol use disorders (Jakubczyk et al., 2018), the current findings show that both factors are relevant to impulsive spending and appear to explain a link between impulsive spending and childhood trauma.

A possible explanation as to why emotion dysregulation acts as a mediator between ACEs and impulse spending is that an exposure to ACEs in early childhood can have long term psychological impacts, leading to negative mood (Jaworska-Andryszewska & Rybakowski, 2019). As previously discussed, Gardner and Rook (1988) found that individuals who are susceptible to impulse spending often engage in the behaviour to improve their mood. The inability to tolerate negative emotions may lead to maladaptive behaviours and coping mechanisms.

Impulsivity may act as a mediator between ACEs and impulse spending because exposure to trauma in childhood increases the tendency to act impulsively (Shin et al., 2018). Researchers have proposed a number of potential reasons and mechanisms that may explain why childhood trauma can lead to elevated impulsivity; from an evolutionary perspective, childhood maltreatment is likely to lead to a lack of resources and, therefore, a rush to move forwards quickly in one’s life to obtain access to resources (Liu, 2019). Disrupted neurological development may also explain this relationship (Liu, 2019); for example, those with childhood trauma show differing electroencephalography brain activity during experimental impulsivity paradigms (Kim et al., 2018).

Clinically, the present results imply that psychological therapies should seek to reduce impulsivity and improve emotion regulation to mitigate impulse spending in those with histories of early adversity and childhood trauma. Mindfulness reduces trait impulsivity in those with substance use issues (Davis et al., 2019), and a brief mindfulness intervention has been shown to reduce impulsivity in experimental conditions (Dixon et al., 2019). However, the literature is inconsistent, with other research showing that an 8-week mindfulness course did not reduce impulsivity (Korponay et al., 2019). Dialectical behaviour therapy has been shown to improve emotion regulation (Neacsiu et al., 2014; Rozakou-Soumalia et al., 2021) and reduce impulsive behaviours (Jamilian et al., 2014). Future research should adapt these therapies to see if they can reduce or prevent impulsive spending in those who are at high risk due to early childhood adversity.

While this research has important implications for those with impulsive spending behaviours, the study is limited by the cross-sectional and correlational design and retrospective reports of childhood trauma, which preclude causality in the mediation analyses; future researchers should examine the present associations longitudinally or experimentally. Nevertheless, research has shown good test–retest reliability of the Childhood Trauma Questionnaire in clinical populations (Shannon et al., 2016). The sample was predominantly female and of White ethnicity, limiting the generalisability of the findings. Reliance on self-report measures could have resulted in socially desirable responses. However, due to the personal nature of the study, it would be difficult to conduct without some aspect of self-report; future research may seek to incorporate reports from others (e.g., on spending behaviours) to see if these align with self-reports.

Regarding the sample, there was a high proportion of individuals with bipolar disorder and other mental health conditions, likely because this study was advertised by Bipolar organisations and probably appealed to this group given the high prevalence of impulsive spending within this condition (Fletcher et al., 2013; Richardson et al., 2017). The mediation analyses separated by clinical status showed that impulsivity remained a mediator in both clinical and non-clinical participants, though emotional dysregulation was only a mediator in the non-clinical sample. This is surprising given that emotional dysregulation is common in clinical populations including mood and anxiety disorders (De Prisco et al., 2022; Hofmann et al., 2012). It may be that, as the clinical group are more emotionally dysregulated, there was less variance in the clinical sample on this variable, meaning that mediation could not be shown statistically. Another possibility is that impulsivity is a stronger predictor within the clinical sample; research shows that those with bipolar disorder have elevated levels of impulsivity even outside of acute mood episodes (Newman & Meyer, 2014).

## Conclusion

Overall, exposure to childhood trauma is associated with increased impulsive spending in adulthood. Childhood trauma increases impulsive spending via increased general levels of impulsivity and emotion dysregulation; these mechanisms are therefore likely to be important targets in psychological interventions to reduce impulsive spending in those exposed to childhood trauma. Further evidence is required to establish causality, and replication of the observed effects in more representative groups will strengthen the findings.

## Data Availability

Data are available from the corresponding author upon reasonable request.
